# The Hydrophobic Effect in Solute Partitioning and Interfacial Tension

**DOI:** 10.1038/srep19265

**Published:** 2016-01-27

**Authors:** Meyer B. Jackson

**Affiliations:** 1 Department of Neuroscience University of Wisconsin - Madison, 1111 Highland Ave, Madison, WI 53705.

## Abstract

Studies of the partitioning of hydrophobic solutes between water and nonpolar solvents provide estimates for the energy cost of creating hydrophobic-water contacts. This energy is a factor of three lower than the work of adhesion derived from interfacial tension measurements. This discrepancy noted by Tanford in 1979 is widely viewed as a serious challenge to our understanding of hydrophobic interactions. However, the interfacial energy of a water-alkane interface depends on chain length. A simple analysis of published data shows that the loss of rotational freedom of an alkane chain at an interface accounts quantitatively for the length-dependent contribution to interfacial tension, leaving a length-independent contribution very close to the free energy of transfer per unit of solvent accessible surface area. This analysis thus clarifies the discrepancy between the thermodynamic and interfacial tension measurements of hydrophobic interaction energy. Alkanes do not loose rotational freedom when transferred between two different liquid phases but they do at an interface. This reconciles the difference between microscopic and macroscopic measurements. Like the partitioning free energy, the work of adhesion also has a large entropy and small enthalpy at 20 ^o^C.

Both thermodynamic partitioning and interfacial tension measurements can be used to study the energetics of hydrophobic interactions. Thermodynamic measurements of free energies of solute transfer between water and nonpolar solvents provide a microscopic/molecular estimate of the energy cost of exposing nonpolar molecules to water. For a series of molecules, plots of free energy of transfer generally display a linear dependence on molecular surface area, or solvent accessible surface area, with slopes falling in a range of 17–31 cal/Å^2^ 1,2,3. The surface tension of a hydrocarbon-water interface also reflects the energy cost of exposing hydrocarbon to water. These experiments provide the work of adhesion, calculated as the sum of the water-vapor and hydrocarbon-vapor interfacial tensions, minus the hydrocarbon-water interfacial tension. The work of adhesion is a macroscopic measure of the energy cost of exposing a hydrophobic surface to water. A generic value widely cited for the work of adhesion of hydrocarbon-water interfaces is 50 erg/cm^2^, which equals 71.9 cal/Å^2^. This is roughly 3 times larger than the microscopic energy per unit area determined from thermodynamic measurements.

Tanford made the comparison between macroscopic and microscopic estimates of the free energy of hydrophobic interactions and recognized the difference^4^. The discrepancy has attracted a great deal of attention, and has motivated theoretical work to seek an explanation^2^,^5^,^6^,^7^. Tanford based his analysis on data from hexane^8^ and octane^9^. Systematic studies of interfacial tension at water-hydrocarbon interfaces have been conducted and indicate that the work of adhesion varies with chain length among n-alkanes^10^,^11^. This indicates that interfacial tension data do not provide a unique proportionality constant between energy and area, thus complicating comparisons with thermodynamic estimates. Figure 1 displays a plot of the work of adhesion versus chain length for n-alkanes containing 5 to 16 carbon atoms from a study by Goebels and Lunkenheimer^11^ (the data are plotted in the authors’ units of erg/cm^2^). Aveyard and Haydon^10^ reported very similar measurements, and the numbers from the two studies agree to within a few percent. Goebel and Lunkenheimer prepared purer samples, so their measurements can be taken as more accurate. The work of adhesion ranges from 53 cal/Å^2^ (37 erg/cm^2^) for pentane to 63 cal/Å^2^ (44 erg/cm^2^) for hexadecane. These numbers are smaller than the generic value of 71.9 cal/Å^2^ (50 erg/cm^2^), but are still well above the 17–31 cal/Å^2^ range cited above from solute partitioning data. The variation in work of adhesion with chain length indicates that the interfacial tension depends on conformational degrees of freedom of the alkanes. A small even-odd effect in the n-alkane data was also invoked in support of this view^11^. This factor is completely ignored in the simplest and most widely used analysis of partitioning in terms of solvent accessible surface area. Although efforts have been made to incorporate solute conformation into the analysis of partitioning data^12^, the impact is well below the discrepancy between the microscopic and macroscopic measurements.

This brings out an important difference between the partitioning and surface tension experiments. In contrast to hydrocarbon molecules dissolved in water, hydrocarbon molecules at an aqueous interface are in an anisotropic environment that can limit rotation. Other factors such as the distribution of orientations and distances between alkanes at the interface could also contribute to surface tension. These intermolecular interactions between hydrocarbons at an interface are all irrelevant to partitioning. This suggests that we can clarify the relation between these two experimental approaches by decomposing the work of adhesion into a sum of chain length-dependent and chain length-independent contributions. The chain length-independent contribution reflects water reorganization at the hydrocarbon surface, and can also include intermolecular forces between alkanes and water. The chain length-independent quantity should correspond more closely to thermodynamic measurements of free energy of transfer. The chain length-dependent contribution represents factors such as the rotational free energy of the n-alkane.

If a segment of an alkane has its rotations suppressed at an interface then an m-carbon segment loses α^m−2^ conformations (where α is the ratio of rotational states available to a C-C bond in bulk to that at the interface, counting from a methyl terminus where the first two atoms are irrelevant to rotational freedom). A C-C bond reduced from 3 rotational states in bulk to one at the interface would give α ~ 3. We must also consider the distribution of segment lengths at the interface, and the probability of an m-carbon segment being at the interface would depend on m. For each C-C bond at the interface, the probability of the next bond not rotating out of the interface is taken as β. For example, if the three rotational states of a C-C bond are equally likely we have a value for β of 1/3 for the probability that a chain with atom # j at the interface will also have atom # j + 1 at the interface. This gives β^m−2^ for the probability of an m-atom segment residing at the interface. For an n-carbon alkane, the average number of lost rotations can be written as a sum over the number of lost conformations in an interfacial m-carbon segment times the probability of the interfacial segment having a length of m carbon atoms. This gives the sum of the geometric series (αβ)^m−2^ from m = 2 to n, 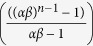. This provides a rationale for the following simple expression for the work of adhesion, W, of a water-alkane interface versus chain length, n.


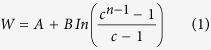


*A* represents the length-independent contribution from water reorganization at the surface as well as any contribution from the hydrocarbon-water intermolecular potential. *B* parameterizes the surface density of the rotational change in entropy, and *C* = αβ.

Eq. 1 fitted the work of adhesion data of Goebel and Lunkenheimer very well (Fig. 1), yielding *A* = 29.6 cal/Å^2^, *B* = 10.9 ± 1.7 cal/Å^2^, and *C* = 0.79 ± 0.03. The large-n asymptote of Eq. 1 is 64.6 cal/Å^2^ (44.9 erg/cm^2^). C < 1 is necessary to recapitulate the observed saturation of Eq. 1 with chain length. The value slightly below one reflects a balance between the number of lost conformations per bond at the interface (α) versus the probability of not rotating out of the interface (β). The inequality β < 1/α indicates that even longer alkane chains have segments at the interface that are relatively short. Within the framework of this model the saturation of W with n suggests that alkanes form an aqueous interface with short segments of many chains rather than long segments of fewer chains. This raises interesting questions about how the interface influences the probability of trans and gauche C-C bonds, as this would have an impact on β. Likewise, α would be lower if the interface can accommodate segments that are not all-trans. Chains with one gauche bond could still lie at an interface but with the surface formed by zig-zag trans bonds not lying flat on the water. A chain with two gauche bonds of opposite angle, such as β-coupled gauche kinks^13^, can also lie approximately in a plane. More detailed analysis of the conformations of alkane chains at a water interface should provide insight into the balance between these various factors.

The quantity *A*, determined from the fit of Eq. 1, can be compared with thermodynamic data, and it does in fact fall within the range of thermodynamic measurements cited above. It is near the high end of the range so factors such as correction for volume fraction and capillary waves would reduce the interfacial tension number somewhat^2^, while alkane conformational flexibility in the partitioning experiments would raise the thermodynamic number^12^. A physical model that treats bond rotations at the interface as well as intermolecular correlations of the hydrocarbon chains more rigorously would of course provide a more accurate framework for decomposing work of adhesion data into various contributions.

By focusing on essential differences between partitioning and interfacial tension measurements, the present analysis resolves the discrepancy between microscopic and macroscopic studies of the hydrophobic effect^4^. This resolution reinterprets an experimental result that had been invoked to support a role for surface curvature^2^,^5^ and a transition in the nature of the hydrophobic interaction with molecule size^6^,^7^. The analysis here suggests comparable energetic costs of water reorganization during exposure to hydrocarbon molecules versus hydrocarbon interfaces. The difference between the microscopic and macroscopic energies likely reflects a difference in alkane bond rotations, which remain free when individual molecules are surrounded by water but experience some restriction at an aqueous interface.

A large entropy at room temperature is an important hallmark of the hydrophobic effect. The enthalpy of hydrophobic partitioning interactions depends strongly on temperature and goes through zero at 22 ^o^C^3^,^14^. Thus, it would be of interest to decompose the work of adhesion of water-hydrocarbon interfaces into entropy and enthalpy. For water-vapor, alkane-vapor, and alkane-water interfaces, temperature dependence data is available from which the entropy per unit area can be determined. The water-vapor interfacial tension in the CRC handbook of Chemistry and Physics is linear with temperature between 10 and 30 ^o^C, and the slope gives an entropy per unit area of −0.152 erg/cm^2^/°C. Temperature dependence data for alkane-vapor and alkane-water interfaces for octane and dodecane^15^ were used to calculate TΔS for the work of adhesion (Table 1).

A comparison of TΔS with ΔG in Table 1 indicates that entropy accounts for at least 90% of the free energy change. Thus, at 20 ^o^C the work of adhesion of alkane-water interfaces like the free energy of solute transfer, arises primarily from a change in entropy and has a very small change in enthalpy. The very strong temperature dependence of hydrophobic interactions arises from a large increase in heat capacity, and like the free energy, the increase in heat capacity scales with solvent accessible surface area^16^,^17^,^18^,^19^. Investigating the temperature dependence of interfacial tension for different hydrophobic molecules may thus provide more insight into the macroscopic manifestations of the hydrophobic effect.

The hydrophobic effect has a major role in determining the energy landscape of biological processes. For example, theoretical calculations have suggested a prohibitive energy cost of exposing the hydrocarbon interior of a lipid bilayer to water during membrane fusion. This motivated the development of the hemifusion-stalk mechanism, which entails virtually no exposure of the core of a lipid bilayer to water^20^. However, this calculation was based on the macroscopic parameter. The hydrocarbon tails of phospholipids are oriented and have restricted rotations in a lipid bilayer, so the chain length dependent term in Eq. 1 is probably irrelevant. This indicates that the microscopic parameter is more appropriate, and an analysis of SNARE-mediated membrane fusion using the microscopic parameter to estimate the energetic cost of transient exposure of the bilayer core to water yields plausible rates of fusion^21^.

## Additional Information

**How to cite this article**: Jackson, M. B. The Hydrophobic Effect in Solute Partitioning and Interfacial Tension. *Sci. Rep.* **6**, 19265; doi: 10.1038/srep19265 (2016).

## Figures and Tables

**Figure 1:**
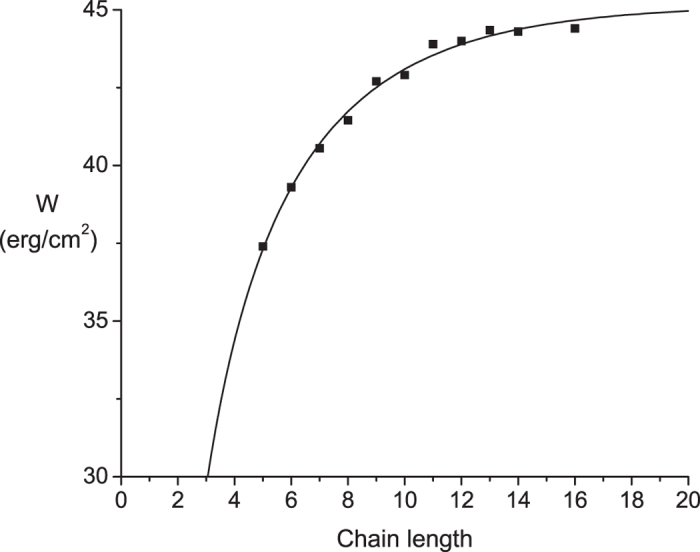
Plot of the work of adhesion (W) versus number of carbon atoms (n) in n-alkanes. Data from Goebel and Lunkenheimer^11^. Eq. 1 was fitted to the data (solid curve) with the computer program R, yielding *A* = 20.6 ± 2.1 erg/cm^2^ (29.6 ± 3.0 cal/Å), *B* = 15.6 ± 2.4 erg/cm^2^ (22.4 ± 3.4 cal/Å^2^), and *C* = 0.79 ± 0.026.

**Table 1:** Interfacial surface tension entropies.

	ΔS Alkane-vapor erg/cm^2^/^o^C	ΔS Alkane-water erg/cm^2^/^o^C	TΔS of adhesion erg/cm^2^	W erg/cm^2^
Octane	−0.095	−0.089	−46.3	42.8
		−0.0835	−47.8	41.4
Dodecane	−0.088	−0.089	−44.2	45.3
		−0.057	−48.0	44

Alkane-vapor data from Aveyard and Haydon^9^,^10^ at 20 ^o^C (upper value) and from linear regression of data from Zeppieri *et al.*^15^ over 10–60 ^o^C (lower value). Work of adhesion (W) from Aveyard and Haydon at 20 ^o^C (upper) and Goebel and Lunkenheimer^11^ at 22 ^o^C (lower). TΔS of adhesion was calculated from the first two columns; the value for water-vapor interfaces was mentioned in the text. The similarities between −TΔS and W indicate that hydrophobic interactions at interfaces are driven by entropy at 20 ^o^C.
